# New evidence from the northern Apennines, Italy, suggests a southward expansion of *Echinococcus multilocularis* range in Europe

**DOI:** 10.1038/s41598-025-91596-7

**Published:** 2025-03-01

**Authors:** Salvatore Andrea Cafiero, Luca Petroni, Luca Natucci, Orlando Tomassini, Thomas Romig, Marion Wassermann, Chiara Rossi, Heidi Christine Hauffe, Adriano Casulli, Alessandro Massolo

**Affiliations:** 1 Ethology Unit, Department of Biology, University of Pisa, Pisa, Italy; 2 Parasitology Unit, Institute of Biology, University of Hohenheim, Stuttgart, Germany; 3 Conservation Genomics Research Unit, Centre for Research and Innovation, Fondazione Edmund Mach, San Michele all’Adige, Italy; 4 National Biodiversity Future Centre (NBFC), S.c.a.r.l. Palermo, Italy; 5 Department of Infectious Diseases, European Union Reference Laboratory for Parasites (EURL-P), Istituto Superiore di Sanità, Rome, Italy; 6 WHO Collaborating Centre for the Epidemiology, Detection and Control of Cystic and Alveolar Echinococcosis, Department of Infectious Diseases, Istituto Superiore di Sanità, Rome, Italy; 7 Faculty of Veterinary Medicine, University of Calgary, Calgary, AB Canada; 8 Chrono-environnement (UMR 6249), CNRS, Université Marie et Louis Pasteur, Besançon, F-25000 France

**Keywords:** *Taenia*, Red Fox, *Vulpes vulpes*, Grey Wolf, *Canis lupus*, Flotation, Faecal PCR, Epidemiology, Parasitic infection

## Abstract

*Echinococcus multilocularis* (*Em*) is a neglected zoonotic cestode circulating among wild canids and voles across the northern hemisphere, and is the aetiological agent of alveolar echinococcosis in humans. The expansion of the European distribution of this parasite has been raising public health concerns in recent decades. We aimed to investigate the occurrence of *Em* and other taeniids in wild carnivore faeces in the Apuan Alps Regional Park and the Monte Pisano mountain chain (northern Tuscany), a few hundred kilometres from the nearest report of *Em* in Italy. Using standard flotation-sieving and nested PCR of partial *cox1* sequences on single harvested Taeniid eggs, we detected several *Em* haplotypes in red foxes and grey wolves in both study areas, suggesting limited genetic diversity. However, these findings could not be confirmed using more sensitive qPCR probes from the same samples. In addition, we report eggs of *Taenia krabbei* and *T. hydatigena* from wolves, *Dipylidium caninum* and *Mesocestoides* sp. in foxes, and *T. polyacantha* and *Mesocestoides litteratus* in both foxes and mustelids. Further investigation of the distribution of *Em* in the northern Apennines is necessary to assess if the presence of this potentially lethal zoonotic parasite is stable and expanding southward.

**Supplementary Information:**

The online version contains supplementary material available at 10.1038/s41598-025-91596-7.

## Introduction

*Echinococcus multilocularis* (*Em*) is a small parasitic cestode of the Family Taeniidae, with a known distribution across the northern hemisphere throughout North America, Asia and Europe^1^. Its complex life cycle involves wild canids (mainly foxes) as definitive hosts, which harbour sexually reproducing adult worms, and small mammals (mainly voles as *Microtus* spp. and *Arvicola* spp.) as intermediate hosts in which the larval stage (metacestode) develops^1^. This parasite is known to cause at least 18,000 new human cases of alveolar echinococcosis (AE) per year^2^, and is considered one of the three most impactful food- and water-borne parasitic zoonoses worldwide^3^.

The expansion^4^ and urbanisation^5,6^ of red fox (*Vulpes vulpes*) populations throughout Europe has been favoured by anti-rabies vaccination campaigns^4^, anthropogenic landscape modification and decreased human persecution^7,8^, that after the 1990s resulted in the increase of the geographic range, prevalence and human exposure to *Em*^9^. This plethora of factors increased environmental contamination and the available parasitic biomass, raising the likelihood that humans would ingest eggs, not only in suburban and rural areas, but even in city centres^10^.

In Italy, the first *Em*-infected foxes were found more than 20 years ago in the Autonomous Province of Bolzano, Italy, near the Italy-Austria border^11^. Since then, *Em* prevalence in foxes was confirmed as 12-14.5% in this autochthonous focus^12,13^. More recently, tapeworm eggs were collected from shepherd dog (*Canis lupus familiaris*) and grey wolf (*Canis lupus*) faeces in the Ligurian Alps, bordering France, and confirmed as *Em* using molecular methods^14^. However, current *Em* distribution in Italy only appears to be stable within the Trentino-Alto Adige/Südtirol Region^15^. Besides *Em*, several other *Echinococcus* and *Taenia* species have been detected in wild carnivores across Italy. These tapeworms involve either sylvatic or domestic trophic cycles, or both^16^. Some species are zoonotic to humans, causing visceral and neural disorders^17,18^, whereas others determine infection in livestock with subsequent economic losses^19^. Cystic echinococcosis, which is caused by the *E. granulosus sensu lato* species complex, is one of the most relevant^20^, with a mainly domestic cycle involving dogs and livestock, and occurring predominantly in southern and insular Italy^21^.

At the same time, known and potential definitive taeniid hosts have been expanding their distributional range during recent decades into different habitat types^4,22^. This is concerning because, while the establishment of taeniids in new areas seems to depend on intermediate host distribution and anthropogenic disturbance of natural habitats, the role of definitive hosts in the introduction of parasites to new areas is relatively unknown. For example, in Europe the grey wolf can potentially play a relevant role in the spread of these parasites, because of their ability to cross ecological barriers and their great dispersal distance even across cultivated land.

This study aimed to investigate the presence of *Em* and other taeniids in an area bordering the Ligurian Apennines, a few hundred kilometres from the western Alps where occurrences of *Em* in wolves and shepherd dogs have recently been reported^14^. More specifically, we aimed to search for Taeniidae infections in wild carnivores (grey wolves, red foxes and mustelids) in the Apuan Alps Regional Park, a protected area of northern Tuscany, and in Monte Pisano, a small area contiguous to the Apennines, but ecologically separated by cultivated lands and small urban settlements, where wolf presence is regularly reported.

## Materials & methods

### Study areas and sample collection

The Apuan Alps are located in northern Tuscany, 5–10 km north of the Ligurian Sea coast. The area is partially isolated from the main Apennines, but is still characterized by a typical alpine environment, with elevation ranging from 200 to 1,947 m above sea level (a.s.l.). The central portion of the chain is protected as the Apuan Alps Regional Park (206 km^2^) and its contiguous area (314 km^2^) (hereafter referred to together as AARP), granting different levels of protection. In contrast, the surrounding valley bottoms are highly anthropized. As a result of its proximity to the sea and morphology of the slopes, the region is one of the rainiest in Italy^23^. Subsequently, vegetation varies dramatically with altitude, from Mediterranean scrub on the hills of the coast, to oak-hornbeam (*Quercus spp.- Ostrya carpinifolia*) associations, widespread chestnut (*Castanea sativa*) woods and beech (*Fagus sylvatica*) at higher elevation; and montane grasslands and bare cliffs are found above the tree line. The carnivore guild in the area includes the wolf, as the only large predator, and seven medium to small-sized species, including the red fox, European badger (*Meles meles*), stone marten (*Martes foina*), pine marten (*Martes martes*), European polecat (*Mustela putorius*), European wildcat (*Felis silvestris*) and common weasel (*Mustela nivalis*). Human presence is ubiquitous and mainly represented by recreational activities inside the park, while marble quarrying and hunting are allowed immediately outside the protected area.

This study area was divided into a grid of 3 × 3 km making a total of 52 cells. Each cell was systematically sampled along a fixed walking route of at least 2 km (2,782.60 ± 888.56 m, Mean ± SD), adapting route path and length to environmental constraints given the harsh terrain (min: 1,401.76 m, max: 6,546.41 m). Each route was walked bimonthly in 2020 and quarterly between January 2021 and April 2023 to collect faecal samples of wild carnivores.

**Fig. 1:**
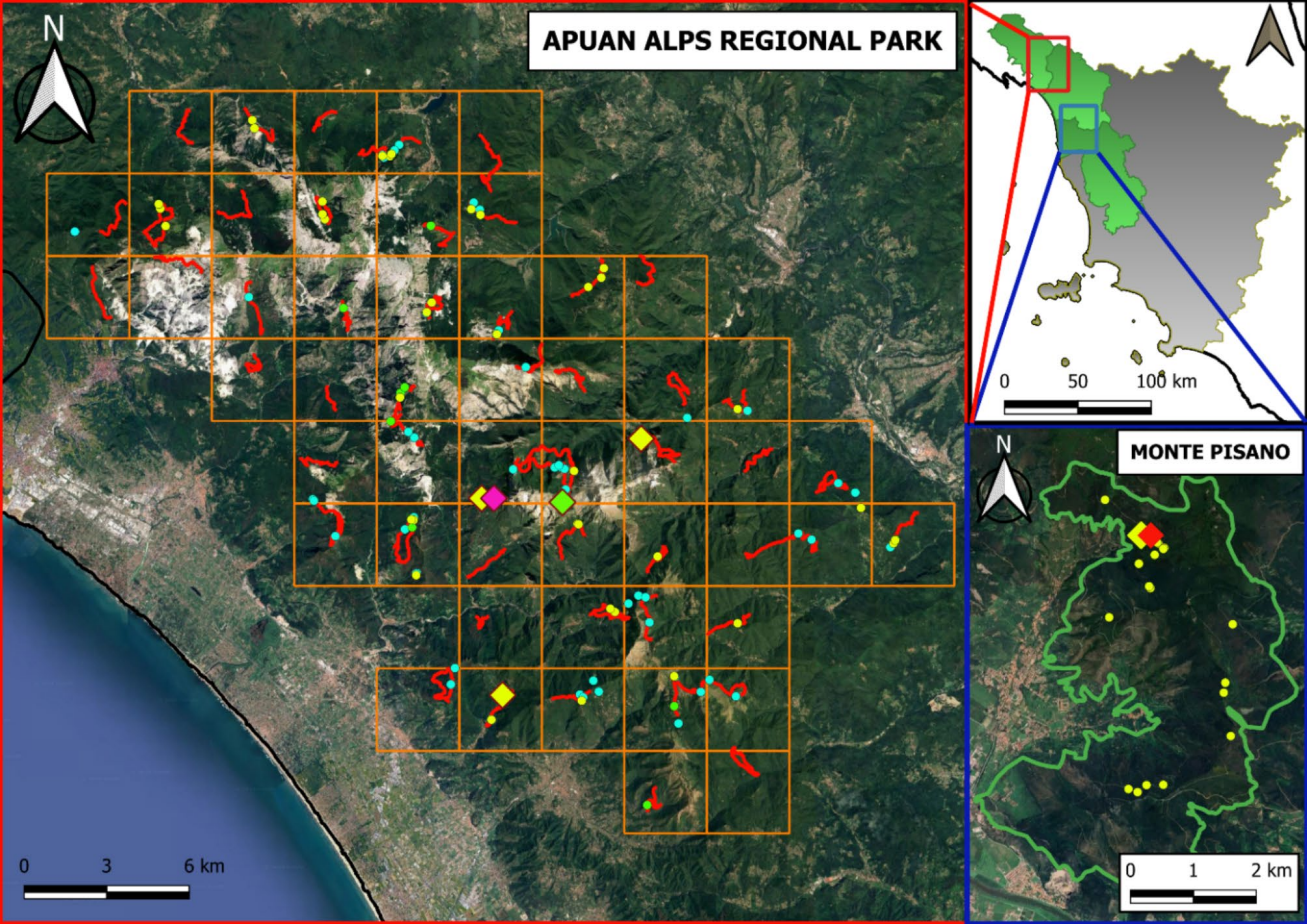
Location of wild carnivore faecal samples (grey wolf in light blue, red fox in yellow and mustelid in light green dots) collected from February 2020 to April 2023 from both the Apuan Alps Regional Park (red box) and Monte Pisano area (blue box), Tuscany (inset map), Italy, for investigating the presence of Taeniidae eggs. The 3 × 3 km grid of AARP is described along with the fixed walking routes (in red). *Cox1* gene profiles of *Em*-positive faecal samples are showed in coloured diamonds: yellow for AARP *cox1*-1 and MP *cox1*-1, purple for AARP *cox1*-2, light green for AARP *cox1*-3 and red for MP *cox1*-2. Partially overlapping diamonds represent co-infection with distinct *cox1* profiles in the same faecal sample. The map was created using QGIS 3.38 software (https://qgis.org/project/visual-changelogs/visualchangelog338/).

In addition to AARP, some opportunistic sampling was carried out in June and July 2021 in an 18 km^2^ wide area of Monte Pisano (MP) as part of another ongoing project^24^ (Fig. 1). The whole MP complex is located about 20 km south of AARP, representing an extension of the Apuan Alps between the Serchio and Arno rivers, separating Lucca and Pisa provinces, with an altitude ranging from 112 to 917 m asl, and a typically Mediterranean climate with humid winters and arid summers^25^. In this fire-prone location, the current vegetation is dominated by introduced chestnut *C. sativa* and maritime pine *Pinus pinaster* forests. The shrub layer is a typical Mediterranean scrub^26^. The carnivore guild includes four Mustelidae species (common weasel, European polecat, stone marten, and European badger), red fox and, occasionally, grey wolf.

For each excrement, GPS position was noted, the faecal pellet was collected following a specific field protocol to avoid contamination and minimize risk to field workers (**Supplementary Material 1**), and stored in a double zip-lock plastic bag. Following a visual inspection of morphometric parameters, each sample was assigned to probable species and to a quality category (1, wet and fresh (deposited 1–2 days before collection); 2, not wet but relatively fresh (2–3 dd); 3, dry but with residual matrix (5–7 dd); 4, very old (> 7 dd); see **Supplementary Material 1** for a detailed description). To avoid potential bias due to sample conditions, only scats belonging to quality categories 1 and 2 were used for parasitological analyses.

All faecal samples were stored at -80 °C for at least five days in order to deactivate taeniid eggs^27^, and, subsequently, at -20 °C until parasitological and dietary analyses. Each fresh faecal sample was split into four portions, from which equal aliquots were taken to form two homogenous subsamples of 1–2 g to be used for two distinct parasitological analyses. Aliquots were summed up to a total of 1 gram per subsample from mustelid and small fox scats or a total of 2 g per subsample from big fox scats and all wolf ones.

### Taeniid eggs harvest, and DNA extraction, amplification and sequencing

A standard flotation and sieving method with 50 and 22 μm mesh size sieves and zinc chloride solution of 1.45 specific gravity^28^ was performed at the Department of Parasitology of the University of Hohenheim, Stuttgart, Germany. The resulting residue was examined under an inverse microscope for taeniid eggs and, where present, 10 to 20 eggs per sample were transferred individually via pipette into a 2 ml Eppendorf tube with 10 µl of 0.02 M NaOH^29^. After heating at 95 °C for 10 min, 1 µl of each lysate was used as template for nested PCR in order to amplify partial mitochondrial *nad1* and *cox1* genes^30,31^ with specific primers (Table 1). If both procedures yielded negative results, a smaller fragment of ~ 200 bp from *nad1* gene was targeted. For the first PCR, a 25 µl reaction mixture was prepared with 10 pmol of each external primer, 200 µM dNTPs, 10 mM Tris–HCl (pH 8.3), 50 mM KCl, 2 mM MgCl_2_, and 0.625 U of Taq polymerase. After amplification, 2 µl of product were transferred to a second PCR with a 50 µl reaction mixture containing the same ingredients as the first PCR, except for 20 pmol of each internal primer. All thermal reactions consisted of an initial denaturation step (95 °C, 5 min), followed by 35 cycles of denaturation (95 °C, 30 s), annealing (50 °C, 30 s), extension (72 °C, 60 s) and a final extension (72 °C, 5 min). Products of approximately 945, 450 and 200 base pairs, respectively, were then sent for Sanger sequencing to Microsynth Seqlab GmbH (Göttingen, Germany).

**Table 1:** Primer pairs used for nested PCR on egg-DNA extracted from wild carnivore faecal samples collected from February 2020 to December 2022 from both the Apuan alps regional park and Monte Pisano area, Tuscany, Italy. F: forward; R: reverse.

Target gene	1st reaction	2nd reaction
*nad1* ^*a*^	F: 5’-TGGAACTCAGTTTGAGCTTTACTA-3’ R: 5’-ATATCAAAGTAACCTGCTATGCAG-3’	F: 5’-TATTAAAAATATTGAGTTTGCGTC-3’ R: 5’-TCTTGAAGTTAACAGCATCACGAT-3’
*cox1* ^b^	F: 5’-TTTGATCGTAAATTTAGTTCTGC-3’ R: 5’-GCAACAACAAATCAAGTATCATG-3’	F: 5’-GTTCTGCTTTTTTTGATCC-3’ R: 5’-GTATCATGTAGAACTTTATC-3’
short *nad1*^a^	F: 5’-TGTTTTTGAGATCAGTTCGGTGTG-3’ R: 5’-CATAATCAAACGGAGTACGATTAG-3’	F: 5’-CAGTTCGGTGTGCTTTTGGGTCTG-3’ R: 5’-GAGTACGATTAGTCTCACACAGCA-3’

^a^ Hüttner et al. 2008^31^; ^b^ Dumendiak et al. 2024^32^.

The flotation residue from the subsample of the *Em*-positive fox faeces was submitted to the European Union Reference Laboratory for Parasites (EURL-P; Rome, Italy) for molecular confirmation.

### *Em* copro-DNA amplification and sequencing

The second faecal subsample was used for molecular screening of *Em*. DNA extraction and amplification were performed at the Animal, Environmental and Antique DNA Platform and the Sequencing and Genotyping Platform of the Fondazione Edmund Mach, San Michele all’Adige, Italy. Whole DNA was extracted from 200 mg of faecal subsample through an automated magnetic bead-based extraction kit (Mag-Bind Stool DNA 96 kit Omega Bio-Tek, USA)^32^, after five freeze-thawing steps to encourage the release of DNA from eggs^33^, with a final elution of 150 µL. Molecular amplification (ViiA 7 Real-time PCR System, ThermoFisher Scientific, Waltham, MA) targeted two different genes with separated protocols and reagents (Table 2): 1) the *Em*-specific 84 bp mtDNA marker *rrnL*^34^ (qPCR protocol following Knapp et al.^35^ with minor modifications). 1 µl of internal control (IAC) plasmid (1000 copies/µl), specifically designed to be amplified by the same *rrnL* primers, was added to evaluate the presence of inhibitors (provided by the ANSES laboratory in Nancy, National Reference Laboratory for *Echinococcus* spp., Malzéville, France); 2 pmol of *rrnL*-probe and IAC-probe were also added to detect the plasmid’s fluorescent signal and the *Em*-DNA amplicon; 2) the *Em*-specific 126 bp fragment of the *nad2* gene (qPCR following Santa et al.^32^ with QuantiNova Pathogen + IC Kit, Qiagen, Germany, following manufacturer’s instructions).

**Table 2:** Primers and probe used for qPCR targeting *RrnL* and *nad2* genes of *Echinococcus multilocularis* in wild carnivore faeces sampled from both the Apuan alps regional park and Monte Pisano area, Tuscany, Italy, from February 2020 to April 2023.

Target gene	Primer/probe	Oligonucleotide sequence (5’-3’)
*rrnL* ^*a*^	rrnL_f	CTGTGATCTTGGTGTAGTAGTTGAGATTT
*rrnL* ^*a*^	rrnL_PCR	GGGGTCAATCACAACAACCC
*rrnL* ^*a*^	rrnL_P	TGGTCTGTTCGACCTTTTTACCCTCCAT
*nad2* ^*b*^	Nad234_F	TTGTTGAGCTATGTAATAATGTGTGGAT
*nad2* ^*b*^	Nad234_R	CATAAATGGAAACAAACCAAACTTCA
*nad2* ^*b*^	Nad234_P	FAM-CTGTGCTATTAGTCTC-MGB-NFQ

^*a*^ Knapp et al. 2016^36^; ^*b*^ Santa et al. 2019^33^.

### Prevalence estimates

Apparent and true prevalences (AP and TP) were estimated according to the Jeffreys^36^ method on EpiTools (Sergeant, ESG, 2018. Epitools Epidemiological Calculators. Ausvet. Available at: http://epitools.ausvet.com.au) and in R (version 4.4.1) using ‘binom’ package and ‘binom.bayes’ function. Sensitivity and specificity of flotation and sieving followed by PCR were 0.548 and 0.934 respectively, as noted by Otero-Abad and colleagues^37^; whereas those for the *rrnL*-specific qPCR were as estimated by Knapp and colleagues^34^ (0.897 and 0.9286, respectively) and Obber and colleagues^13^ (0.83 and 0.97, respectively).

### Sequence analyses

The sequenced taeniid-DNA amplicons were compared with *cox1* and *nad1* sequences present in the NCBI BLAST database (https://blast.ncbi.nlm.nih.gov/Blast.cgi?PROGRAM=blastn&PAGE_TYPE=BlastSearch&LINK_LOC=blasthome). Sequences alignment and analysis were done in GENtle^38^ and MEGA-X^39^ softwares.

## Results

### Flotation, Sieving and PCR

A total of 130 faeces (9 mustelid, 61 fox and 60 wolf; freshness category 1 and 2) was collected between 2020 and 2022 (of which 20 red fox samples from MP) and subsampled for flotation, sieving and nested PCR (Fig. 1). Taeniid eggs were detected and harvested from 49 faecal samples (6 mustelid, 18 fox and 25 wolf) from AARP, and one sample of MP, for a total of 367 and 20 eggs for each sampling area, respectively. Only 128/367 (34.9%) and 2/20 (10%) eggs were successfully PCR-amplified and sequenced. Overall, molecular characterization of eggs was successful in 11.1% (1/9) of the mustelid, 8.2% (5/61) of the fox and 26.7% (16/60) of the wolf scats (Table 3). *E*. *multilocularis* was the only tapeworm detected in MP. In the Apuan Alps, eight eggs were molecularly identified as *Em*, 55 as *T. hydatigena*, 35 as *T. krabbei* and 17 as *T. polyacantha*. Additional 10 eggs belonged to other cestode families, namely 6 *Mesocestoides* sp., 3 *M. litteratus* and 1 *Dipylidium caninum*. *E*. *multilocularis* DNA was identified from 1/41 fox (AP = 0.0244, 95% CI = 0.0026–0.1084; TP = 0.0357, 95% CI = 0.000047–0.0915) and 3/60 wolf (AP = 0.05, 95% CI = 0.0143–0.1274; TP = 0.0574, 95% CI = 0.0088–0.1155) samples from AARP, but only in one out of 20 fox scats (0.05, 95% CI = 0.0054–0.2108; TP = 0.0714, 95% CI = 0.0001–0.1796) from MP (Fig. 1). *T. hydatigena* and *T. krabbei* were detected in wolves, whereas foxes had eggs of *T. polyacantha*, *M. litteratus*, *Mesocestoides* sp. and *D. caninum*. Only one mustelid stool sample had eggs of *M. litteratus* and *T. polyacantha*.

**Table 3:** Number (and percentage) of wild carnivore faecal samples collected in the Apuan alps regional park and Monte Pisano area, Tuscany (Italy), from February 2020 to December 2022 containing parasitic eggs after flotation and Sieving and nested PCR. AARP: Apuan alps regional park; MP: Monte Pisano.

Cestode species detected after floatation and sieving and nested PCR on egg-DNA							
	*Echinococcus multilocularis*	*Taenia hydatigena*	*Taenia krabbei*	*Taenia polyacantha*	*Mesocestoides litteratus*	*Mesocestoides* sp.	*Dipylidium caninum*
Grey wolf	**3 (5.0)**	10 (16.7)	7 (11.7)	0	0	0	0
Red fox (AARP)	**1 (2.4)**	0	0	1 (2.4)	1 (2.4)	1 (2.4)	1 (2.4)
Red fox (MP)	**1 (5.0)**	0	0	0	0	0	0
Mustelid	**0**	0	0	1 (11.1)	1 (11.1)	0	0

Co-infections with multiple species occurred in five cases (Table 4). The sieving process prevented the detection of eggs from many other parasite species. Nonetheless, we detected several other canid parasites, such as Ancylostomidae and *Eucoleus* spp. (both *E. boehmi* and *E. aerophilus*), Strongylidae larvae, in addition to *Sarcocystis* sp. oocysts and some *Demodex* sp. mites (possibly originating from carnivore prey). Their species determination was beyond the goal of this project.

**Table 4:** Co-infections of different cestodes of wild carnivores collected from February 2020 to December 2022 from both the Apuan alps regional park and Monte Pisano area, Tuscany, Italy. *Nad1*+: positivity after PCR on *nad1* gene; *cox1*+: positivity after PCR on *cox1* gene; /: no species detected.

Species	ID	*N* extracted eggs	nad1+	nad1 species	cox1+	cox1 species
Grey wolf	AA.9	25	0	/	2 2 1	*T. hydatigena* ***E. multilocularis*** *T. krabbei*
Grey wolf	AA.80	12	3	*T. krabbei*	2 1	*T. krabbei* *T. hydatigena*
Grey wolf	AA.99	23	1	***E. multilocularis***	9 1	*T. hydatigena* ***E. multilocularis***
Red fox	AA.23	16	0	/	10 3	*T. polyacantha* ***E. multilocularis***
Mustelid	AA.52	10	2	*M. litteratus*	7	*T. polyacantha*

Molecular analyses (nested PCR) conducted at the EURL-P (Rome, Italy) on the flotation residual from the *Em*-positive fox (ID AA.23), confirmed three isolated eggs belonged to *Em*.

### Molecular identification and sequencing of *Em* eggs

**Fig. 2:**
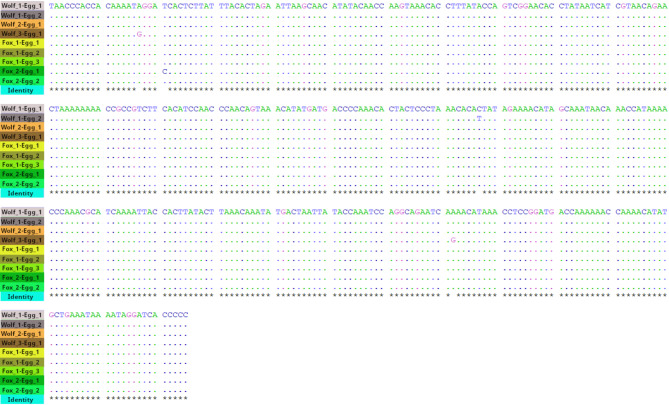
Alignment of nine 355 bp sequences of *cox1* gene amplified from nine different *Echinococcus multilocularis* eggs. The three wolf haplotypes and the Fox_1 haplotype originate from Apuan Alps Regional Park (AARP), while the Fox_2 haplotype originates from Monte Pisano (MP) area, Tuscany, Italy.

The characterisation of *Em* eggs harvested from faecal samples was based on the *cox1* gene, except one case where the short *nad1* fragment was analysed. Nine 355 bp *cox1* sequences (seven from AARP isolated from three wolves and one fox, and two from MP isolated from 1 fox) were aligned. Four *cox1* haplotypes were identified (Fig. 2). The most common (6/9) genetic variant (PQ479227) was isolated from both wolves and foxes and has already been detected in many European countries, including France (e.g. OQ599957) and Switzerland (e.g. MT461411), but also in North America. The three other sequences differed from the first one by one or two nucleotide substitutions and are new haplotypes (GenBank Accession Numbers: PQ479228, PQ479229, PQ479230). Two haplotypes each were identified in one wolf (PQ479227, PQ479228) and one fox (PQ479227, PQ479230), respectively.

### qPCR of Em DNA in carnivore faeces

A total of 168 faeces (11 mustelid, 77 fox and 80 wolf) was collected between February 2020 and April 2023 (of which 20 red fox samples from MP) and subsampled for molecular analyses. Neither qPCR protocols yielded positive results, even after dilution of extracted DNA up to 1:5 and 1:10. According to this, AP 95% CI estimates ranged from 0 to 0.0308 (using both Knapp et al. and Obber et al. sensitivity and specificity values), whereas TP 95% CI estimate ranged from 0 to 0.0236 in wolves. With regards to fox faeces, AP 95% CI estimates ranged from 0 to 0.0429 following both reference parameters, while TP 95% CI estimate from 0 to 0.033.

## Discussion

Our study aimed to investigate the presence of *Em* in the Apuan Alps, an area contiguous to the southernmost range of this parasite in Italy, recently found in the Ligurian Maritime Alps. The detection of *Em* in this new area represents, to the best of our knowledge, the first report of *Em* in the Apennines and represents a southern expansion of *Em* in Italy, and therefore, in Europe. More importantly, our data indicate local *Em* circulation in several canid species.

During recent years, the occurrence of taeniid cestodes has been studied in northern^40–43^ and central^44–47^ Italy without ever detecting *Em* in wild or domestic canids, suggesting that the Apennine mountains are an unsuitable habitat for the parasite’s life cycle^48^. On the other hand, previous studies were carried out in different seasons and with different sampling efforts, using diagnostics with lower sensitivity and specificity, which might partially justify the *Em*-negative reports in the past^49,50^. Therefore, we cannot confirm that our findings are due to the recent spread of this parasite, or that *Em* is more widespread than previously believed.

Host guild could also account for current *Em* distribution in this area. For example, according to Guerra et al.^51^, the presence of the common vole, *Microtus arvalis*, one of two main intermediate hosts across Europe^52^, contributes significantly to the stability of the Alpine distribution of *Em*; therefore, the absence of this host species in our study area might explain why the presence of *Em* is not stable. In contrast, it has also been suggested that it is the whole prey assemblage of the definitive host diet that supports the establishment of the *Em* life cycle, not just a single species^53^. Given these conflicting views, it is apparent that parameters driving *Em* distribution need to be more thoroughly examined. In the meantime, it should be recognized that spillover to new intermediate hosts^54^ is a potential factor for parasite range spread that cannot be ruled out. In addition, numerous competent intermediate hosts are present along the Apennines, e.g. additional *Microtus* spp. (Simoncini et al., submitted), *Clethrionomys glareolus*,* Chionomys nivalis*,* Mus musculus domesticus*,* Rattus norvegicus*,* Apodemus sylvaticus*,* Myocastor coypus* and *Lepus europaeus*)^55,56^, although more work is needed to properly assess their absolute and relative contribution to fox and wolf diet, and to study potential dilution effects as reported in other systems for *Em*^57,58^.

In previous publications, the Italian Alps were deemed to be the southern edge of European *Em* distribution^9^, primarily because *Em* was only known to occur in Italy in two Alpine foci as described above: one in the Autonomous Province of Bolzano from the northeastern Alps^15^ and one more recently reported in the Ligurian Maritime Alps in the north-west^14^. However, the two foci may have established at very different history: the Trentino-Alto Adige/Südtirol Region in the north-east represents an autochthonous focus^15^ and has been repeatedly confirmed over two decades. On the contrary, the Ligurian Maritime Alps focus^14^ may be the result of a recent introduction by wolves migrating from France^59^; in fact, its establishment has yet to be confirmed as *Em* has not been reported thus far in the main definitive host, the red fox. We cannot exclude, though, that other factors might be acting to modify *Em* distribution, such as climate change and land use modifications which are deemed to affect the complex life cycle of *Echinococcu*s species through egg survival and distribution^60^.

The infections detected here by analysing *Em* eggs from both fox and wolf faeces could not be confirmed by qPCR, possibly because faecal DNA was of low quantity and/or quality. Given the number of internal controls and, more importantly, the detection of *Em* DNA sequences with distinct nucleotide substitutions, we believe that contamination of egg samples is highly unlikely.

The diversity of *Taenia* spp. in wolves and foxes resembled that reported for other regions of Italy where it has been suggested that the interspecific distribution of cestode species hosted by wolves and small to medium carnivores (foxes and mustelids) could be explained by their distinct diets. For example, both *T. hydatigena* and *T. krabbei* were detected in wolf populations from Tuscany and neighbouring regions^14,40,46^ that prey on wild and domestic ungulates like most wolves across Europe^61,62^. In particular, *T. krabbei*, has been recently genetically distinguished from *Taenia ovis*^63^, and also found to infect specific intermediate hosts: *T. krabbei* circulates in wild cervids (e.g. roe deer)^64,65^, whereas *T. ovis* is limited to domestic bovids, mainly sheep and goats. *Mesocestoides* spp. and *T. polyacantha* were isolated here from fox scats, confirming previous studies from the northern Apennines^41,42^, as well as in one mustelid sample. The intermediate host range of *T. polyacantha* includes several vole species such as *Microtus oeconomus*, *M. arvalis* and *C. glareolus*^16^, whereas the *Mesocestoides* spp. life cycle involves a wider spectrum of small prey, e.g. amphibians, reptiles, birds and small mammals^66^. *Mesocestoides* spp., including *M. litteratus*, occur in several wild and domestic hosts^67^, but cryptic species are yet to be defined^68^. Our study did not detect other tapeworm species of interest for livestock and humans, including aetiological agents of coenurosis (e.g. *Taenia serialis* and *Taenia multiceps*)^18^. Instead, *D. caninum*, a flea-transmitted tapeworm that generally occurs at low prevalence in red foxes from Europe^69^ and Italy^12,41,45^, was found in AARP in one fox sample. Unlike other studies from Italy^70^, however, neither *Mesocestoides* spp. nor *D. caninum* were identified in wolf faecal samples in this study. Unexpectedly, no *E*,* granulosus s. l.* complex parasites were identified in wolves either, either from egg or qPCR detection methods. This is in contrast with previous results from northern and central Italy^14,46^, which include areas with high anthropic impact^47,71^. A lower consumption of domestic livestock in our study areas might be a reason^72^, but this hypothesis needs additional investigation.

## Conclusions

Our results suggest that the *Em* range may be expanding along the Apennine mountain chain, south of the previously known limit in the Italian (Ligurian) Alps. However, caution is recommended since the accepted gold standard for *Em* confirmation from faecal samples (qPCR) was negative in all samples collected (even our cox1-positive ones), and because we cannot confirm whether these findings indicate the establishment of a new autochthonous focus with a stable circulation in the area, or are due to the temporary presence of the parasite. Despite this, the finding of *Em* eggs in both wolf and fox in these newly sampled areas is of public health concern, and we encourage local government agencies to establish appropriate surveillance for *Em* in the main definitive host (fox), but also in sheep dogs and wolves. Within the context of future epidemiological studies, the genetic characterization of further *Em* haplotypes could elucidate the dispersion pattern of and the phylogenetic relationships with other European isolates^73^, including those in Liguria.

## Electronic supplementary material

Below is the link to the electronic supplementary material.

Supplementary Material 1

## Data Availability

The Echinococcus multilocularis partial cox1 sequences detected in both wolf and fox samples, which have been generated and analysed during the current study, will be available within a few days from the scheduled release date (April 1st 2025) in the GenBank repository (GenBank Accession Numbers: PQ479227, PQ479228, PQ479229, PQ479230) [https://www.ncbi.nlm.nih.gov/genbank/]. These sequences will be simultaneously made available to other INSDC databases, the European Nucleotide Archive (ENA) and the DNA Data Bank of Japan (DDBJ).If anyone wants to request data from this study, the corresponding author Massolo A. should be contacted.
