# Supplementary material for: New evidence from the northern Apennines, Italy, suggests a southward expansion of Echinococcus multilocularis range in Europe
Source: Sci Rep. 2025 Mar 1;15:7353. doi: 10.1038/s41598-025-91596-7 (PMC11873164; doi:10.1038/s41598-025-91596-7)
Supplement: Supplementary file 1 — Supplementary Material 1 [file 41598_2025_91596_MOESM1_ESM.docx]

SUPPLEMENTARY MATERIAL

**Supplementary Material 1.** Field protocol for wild carnivore excrement collection in Apuan Alps Regional Park (AARP) and Monte Pisano (MP) study areas, northern Tuscany, Italy. The classification criteria for determining the quality of faecal samples are described in 3.2.8.

**Supplementary Material 1**. Protocol for field collection of wild carnivore faecal samples.

Materials

- Disposable latex gloves
- Dog poop bags (or similar-sized plastic freezer bags)
- Double zip-lock plastic bags (medium size)
- Extra-large zip-lock plastic bags
- FFP2 or FFP3 mask
- Genotubes
- GPS device or smartphone with GPS App
- Hand sanitizer (gel or disinfectant)
- Hermetic plastic box
- Paper sheets
- Pencil
- Plastic bags

**1.** Coordinates: note the coordinates of the collection point through a GPS device or a smartphone App.

- 1. Recommended smartphone Apps: OruxMaps (Android), Avenza Maps (Android/iOS).
  2. Coordinates should be noted in WGS84/UTM32N (EPSG: 32632) reference system

**2.** Field card annotation: note all the required information in the appropriate field card.

**2.1.** The field card is composed by a table and a legend for all the possible information you may need to compile it.

**3.** Paper sheet: note the excrement’s ID code and the eventual Genotube code on a paper sheet.

**3.1.** Write with pencil only.

**3.2.** The excrement ID code is generated as follows:

**3.2.1.** A letter for the first name and a letter for the last name of the operator collecting the sample.

**3.2.2.** A dash “ - ”.

**3.2.3.** The three-letter code identifying the cell in which the sample is collected.

**3.2.3.1.** In case a sample is collected outside the usual systematic grid use the code OUT.

**3.2.4**. Date in Italian format GGMMAA.

**3.2.5.** A dash “ - “.

**3.2.6.** A progressive number identifying the nth excrement collected on the current trail independently from the species.

**3.2.7.** A letter for the genus and a letter for the species name

**3.2.7.1.** *Canis lupus* CL

*Martes martes*, *M. foina*, *Mustela putorius* MU

*Meles meles* MM

*Mustela nivalis* MN

*Sus scrofa* SS

*Vulpes vulpes* VV

**3.2.8.** A number from 1 to 4 identifying the quality of the faecal sample collected.

**3.2.8.1.** Class 1 is defined as fresh samples with a high matrix content and mucus present also on the upper portion of the excrement; adult flies may have found the pellet (usually, excrements deposited 0-48 h prior to sampling).

**3.2.8.2.** Class 2 is defined as relatively fresh samples with a high matrix content; mucus is dried on the exposed surfaces but faecal pellet is still damp underneath if flipped with the help of a stick; mold is absent; adult flies may be abundant (usually, excrements deposited between 2 days to 5 days prior to sampling, depending on environmental conditions).

**3.2.8.3.** Class 3 is defined as older excrements that have a portion of residual matrix; the general structure of the sample is still conserved; they are (almost) completely dry; molds and mosses can emerge and coleopteran and dipteran detritivores feed on the remains (generally, faecal pellets deposited more than 5 days prior to sampling, depending on environmental conditions).

**3.2.8.4.** Class 4 is associated with old faecal pellets that are disaggregated; they are completely dry or completely disintegrated; molds, mosses and detritivores are thriving or have already completed their reproductive cycle (generally, excrements deposited more than one to two weeks prior to sampling, depending on environmental conditions).

**3.2.8.4.1.** For Class 4 samples, only the ones that maintain the general structure of the sample should be collected (and can be used for morphological diet analysis), to reduce the risk of misidentification and under-representation of the food items subject to faster degradation.

**3.2.9.** For excrements collected during the intensive genetic sampling add the letter “g” at the end of the code.

**3.2.10.** For excrements collected opportunistically during other activities add the letter “o” at the end of the code.

**3.3.** The Genotube code is a seven-digits numerical code reported both on the cap and the side of the Genotube.

**4.** Place the paper sheet inside a double zip-lock plastic bag.

**5.** Wear your personal protective equipment (D.P.I.): disposable latex gloves and FFP2/3 mask.

**6.** Sample collection:

**6.1.** Proceed with the collection of a genetic sample with the Genotube for fresh excrements only (quality 1 or 2).

**6.1.1.** Quality 1: rub the swab somewhat vigorously over multiple portions of the external surface of the excrement, especially where there is mucus; avoid inserting the swab into the faecal sample. If the faecal pellet is very soft, the swab can be ‘dabbed’ over the outer surface.

**6.1.2.** Quality 2: if suitable for genetic sampling but too dry on the upper surface, prior to swabbing, turn the sample over with a twig (always wearing the personal protective equipment) and swab the damp lower surface.

**6.1.3.** Quality 2: if suitable for genetic sampling but dry on all surface areas, wet it with a small quantity of distilled water or water from a sealed plastic bottle and proceed with swabbing.

**6.1.4.** Maintain the Genotube at ambient temperature, in a dry place away from direct sunlight and heat sources until it can be archived (-20 °C).

**6.2.** Collect the whole excrement (if possible) using a dog poop bag or a similar plastic bag.

**6.2.1.** Use your gloved hand inside the dog poop bag to pick up the excrement.

**6.2.2.** Turn the dog poop bag inside out over the excrement, taking care not to contaminate the outside of the bag with faecal matter.

**6.2.3.** Close the dog poop bag with a knot.

**7.** Put the knotted dog poop bag inside the double zip-lock plastic bag and close it.

**8.** Place the collected samples in an extra-large zip-lock bag (hereinafter ‘carry bag’).

**8.1.** All samples collected must be placed in the carry bag during the field activities.

**9.** Remove the disposable latex gloves by flipping the righthand glove inside out, and using the inside of the righthand glove to remove the lefthand glove (inside out). This way nigher hand touches the outside of either glove. Place both in another plastic bag (hereinafter ‘gloves bag’).

**9.1.** Place the gloves bag in the carry bag and seal the latter with a knot.

**10.** Hang the carry bag (closed) outside your backpack or put it inside the backpack in pocket separate from your personal belongings.

**11.** Sanitize your hands with gel or disinfectant.

**12.** The carry bag with all its contents (i.e., samples and gloves bag) must be conserved in a sealed plastic/styrofoam box when being transported in a vehicle.

**12.1.** In warmer ambient temperatures, put ice packs in the plastic/styrofoam box to avoid degradation of the DNA in the samples. Set a portable fridge to 4 °C.

**13.** Sample inactivation: store the samples at -80 °C for 5-7 days to inactivate *Em* and other Taeniids.

**14.** At the end of the inactivation period, samples can be more safely handled for diet and/or parasitological analyses and can be stored at -20 °C. Bacteria and viruses may not necessarily be inactivated so all faecal samples of all species must be handled (and bags opened) in at least BSL2 conditions with suitable DPI.
